# Molecularly targeted therapy for Kaposi's sarcoma in a kidney transplant patient: case report, "what worked and what did not"

**DOI:** 10.1186/1471-2369-8-6

**Published:** 2007-03-27

**Authors:** Patricia Volkow, Juan W Zinser, Ricardo Correa-Rotter

**Affiliations:** 1 Infectious Diseases Department, Instituto Nacional de Cancerología, San Fernando 22, México DF 14050, México; 2 Medical Oncology Division, Instituto Nacional de Cancerología, San Fernando 22, Mexico DF 14050, Mexico; 3 Department of Nefrology and Mineral Metabolisim, Instituto Nacional de las Ciencias Médicas y la Nutrición Salvador Zubirán, Vasco de Quiroga 15, Mexico DF 14000, Mexico

## Abstract

**Background:**

Imatinib is a tyrosine-kinase inhibitor; for which there is limited information regarding its effects on AIDS Kaposi's sarcoma and none in patients with transplant-associated Kaposi's sarcoma. Sirolimus, an immunosuppressive drug used for kidney transplant, exhibits antiangiogenic activity related to impaired production of VEGF (vascular endothelial growth factor), clinical benefit has been reported in Kaposi's sarcoma associated with renal graft.

**Case Presentation:**

Here we report a case of an 80 year old male, who developed Kaposi's Sarcoma nine months after receiving a living non-related donor kidney transplant at age 74. Three years after treatment with different chemotherapeutic agents for progressive cutaneous Kaposi's Sarcoma with no visceral involvement, he was prescribed Imatinib (200 mg/day for two weeks followed by 400 mg/day) after four weeks of treatment he developed anasarca, further progression of KS and agranulocytosis. Imatinib was discontinued and there was significant clinical recovery. One year later his immunosuppressive therapy was changed to Sirolimus and regression of the Kaposi's sarcoma occurred.

**Conclusion:**

The lack of benefit and severe toxicity associated with the use of Imatinib in this patient should alert clinicians of potentially adverse consequence of its use in patients with transplant associated Kaposi's sarcoma. On the other hand the positive response seen in this patient to Sirolimus even after a long evolution of Kaposi's sarcoma, multiple chemotherapy regimens and extensive cutaneous disease further suggest it therapeutical utility for transplant associated Kaposi's sarcoma.

## Background

Since the identification of Human Herpes 8 (HHV-8) in 1994, [1,2] there have been great advances in the understanding of the pathogenesis of Kaposi's sarcoma (KS). The better understanding of autocrine and paracrine factors in the proliferation and differentiation of KS cells [3] has provided a potential benefit derived from targeted therapy, a therapeutic field which has achieved an enormous development over the last decade. There are few reports regarding novel treatment options for KS, this includes the use of Imatinib in AIDS KS [4] and the use of Sirolimus in renal transplant KS patients [5].

Imatinib is a tyrosine-kinase inhibitor that induces apoptosis in Bcr-Abl positive cell lines, platelet-derived growth factor positive cells and c-kit positive gastrointestinal stromal cells. Limited information exists regarding its effects on KS cells [4]. Though the drug safety profile is good it can induce severe adverse events [6]. Because Imatinib is metabolized through the CYP3A3 enzyme it can result in adverse drugs interaction. Its safety in patients with renal-insufficiency or kidney graft is unknown.

HHV-8 up-regulates the vascular endothelial growth factor (VEGF) receptor Flk-1/KDR in endothelial cells. In vitro infection of human primary endothelial cells with HHV-8 causes long-term proliferation and survival of cells. Blocking the interaction between VEGF and Flk-1/KDR can abolish VEGF induced proliferation. Sirolimus an immunosuppressive drug used in kidney transplantation [5], exhibits antiangiogenic activity related to impaired production of VEGF and limited proliferative response of endothelial cells to stimulation by VEGF, therefore inhibiting KS progression.

Here we report a case of a kidney transplant patient who after unsuccessful treatments with different chemotherapeutics was given Imatinib for refractory and extensive cutaneous KS that resulted in severe toxicity and no clinical benefit. In contrast shifting the patient's immunosuppressive maintenance therapy to Sirolimus led within one year to over 80% regression of the KS and is still showing sign of regression (Table 1)

**Table 1:** 

Date	Event
August 2000	End stage renal disease started on hemodyalisis (79 years old male).
March 2001	Living non-related donor kidney transplant.
November 2001	Diagnosed with Kaposi's Sarcoma.
	Three year chemotherapy (vincristine-belomycin) (liposomal adriamycin)
June 2004	Imatinib
July 2004	Agranulocitosis, anasarca increase in creatinine.
February 2005	Placitaxel (developed allergic rash).
April 2005	Change immunosuppressive therapy for Sirolimus.
November 2006	Persistent Kaposi's Sarcoma regression, still on Sirolimus.

## Case Presentation

The patient is an 80 year old male with diabetes mellitus and hypertension, who at age 74 (March 2001) had received a living non-related donor kidney transplant. At that time he was discharged with a double immunosuppressive therapy consisting of prednisone 20 mg/day and mophetil mycophenolate 1.5 gr/day. No calcineurin inhibitor was administered. His prednisone dose was further decreased to 5 mg/day and mophetil mycophenolate doses sustained. In November 2001 he developed cutaneous purple elevated lesions in his lower limbs that were diagnosed as KS by skin biopsy. He received 19 administrations of vincristine (1 mg) and bleomycin (15 mg) weekly that resulted in flattening and fading of the lesions. Treatment was discontinued for two months, and KS recurred, with pain and edema in both legs.

On July 2002, with an almost 100% involvement of his lower limbs skin with KS but no visceral involvement he received 50 mg of liposomal-adriamycin. In addition, he was then prescribed valganciclovir 450 mg BID (corrected for creatinine clearance) [7] and three additional administrations of liposomal-adriamycin, up to December 2002. He showed 40% response again with early relapse. On January 2003 the patient developed disseminated herpes zoster, which was treated with IV acyclovir.

**Figure 1:**
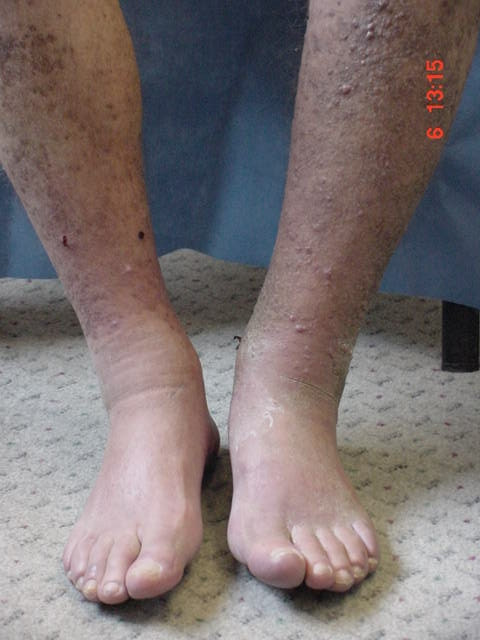
Kaposi's sarcoma progressions after chemotherapy withdrawal, both lower limbs were completely covered with Kaposi's Sarcoma (June 2003).

In May 2003 KS progression was observed and received radiotherapy with no benefit Figure 1. In August 2003 he started oral etoposide at 50 mg QD for two weeks followed by two weeks off until May 2004 achieving a very slow 50% response. Tolerance was good and serum creatinine remain stable at around 1,4 mg/dl. Mophetil mycophenolate dose was reduced to 1 g/day and prednisone to 2.5 mg/day.

**Figure 2:**
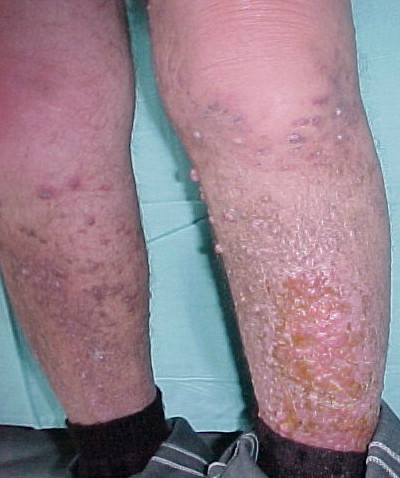
Patient developed anasarca with rapid progression of Kaposi's Sarcoma after four weeks of taking Imatinib (June 2004).

On May 2004 the patient requested a second opinion and was prescribed Imatinib 200 mg qd for two weeks followed by 400 mg qd. After four weeks of Imatinib, his health deteriorated rapidly, he developed anasarca, worsening of KS, his serum creatinine increased from 1.4 mg/dl to 2.3 mg/dl and he developed grade 4 granulocytopenia (Figure 2). The patient was hospitalized with fever and was started on IV antibiotics and daily G-CSF for four days after which his WBC count normalized. Two weeks later the creatinine level returned to 1.5 mg/dl. On February 2005 he was started on 30 mg of paclitaxel IV every three weeks. After three courses with no clinical benefit he developed a skin rash that prompted discontinuation.

**Figure 3:**
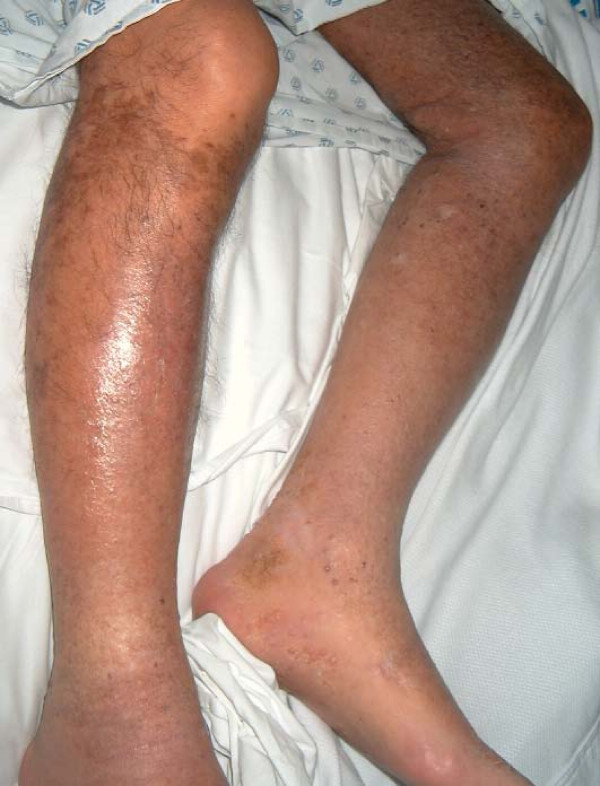
Extensive regression of Kaposi Sarcoma after immune-suppression was changed to Sirolimus, (14 months later), he only has hyperpigmented areas at knee levels, skin looks thin and delicate and easily bruised (July 2006).

On April 2005, he was started on Sirolimus 2 mg a day, and after 8 weeks escalated to 4 mg a day. Three weeks later mophetil mycophenolate and prednisone were discontinued. Sustained and significant KS regression was evident after 16 weeks, but he developed a basal cell carcinoma and pneumonia that required hospital admission; Sirolimus dose was lowered to 2 mg a day. Thereafter his clinical course has been satisfactory with continuous and progressive regression of KS lesions (Figure 3). His serum creatinine has remained under 1.6 mg/dl and he has not developed new infectious episodes.

## Conclusion

KS is a proliferative disease, cytokine mediated, where the presence of the Human Herpes virus 8 is essential for the process, and immunesuppression is a necessary fact [8]. The disease is insidious and with an unpredictable course; when there is no visceral involvement, it is usually not life threatening. [3]

It has been recognized that glucocorticoids have a major role in KS development, [9-11] and their withdrawal may induce regression of KS lesions [12,13]. In vitro they have a synergistic effect with oncostatin, an autocrine element involved in KS-cells proliferation.

A wide variety of therapies have been used for KS, including interferon [14,15] and different chemotherapeutic regimens [16]. Recently Imatinib has been used in KS AIDS patients with short periods of observation [4]. In addition, a recent study demonstrated that Siroliumus inhibited progression of dermal KS in kidney transplanted patients with an outstanding clinical outcome [5].

There is currently no evidence of imatinib effectiveness in transplant associated-KS patients; and there is therefore no basis for its prescription in this group of patients. Moreover, in the case we are reporting, Imatinib not only had no clinical benefit but resulted in life threatening toxicity. In contrast in this patient Sirolimus treatment was associated with extensive regression of severe cutaneous KS even after a long-standing and extended disease, and multiple previous chemotherapy treatments (Table 1). Sirolimus exhibits antiangiogenic activity related to impaired production of VEGF and limiting proliferative response of endothelial cells to stimulation by VEGF, limiting the progression of KS. The KS regression in this case cannot be solely attributed to prednisone withdrawal, since he had been on very low doses of prednisone (2.5 mg qd) months before Sirolimus was initiated. Our results corroborate therapeutic benefit of Sirolimus in kidney graft recipients[5,17,18] both as an immunosuppressant to prevent graft rejection and to induce long standing KS regression.

## Competing interests

The author(s) declare that they have no competing interests.

## Authors' contributions

The three authors of this manuscript: Patricia Volkow, Juan W Zinser, Ricardo Correa-Rotter are treating physicians of the patient reported. All of them have participated in the discussion and writing of the submitted manuscript.

## Pre-publication history

The pre-publication history for this paper can be accessed here:



## References

[B1] Chang Y, Cesarman E, Pessin MS, Lee F, Culpepper J, Knowles DM, Moore PS (1994). Identification of herpesvirus-like DNA sequences in AIDS-associated Kaposi's sarcoma. Science.

[B2] Huang YQ, Li JJ, Kaplan MH, Poiesz B, Katabira E, Zhang WC, Feiner D, Friedman-Kien AE (1995). Human herpesvirus-like nucleic acid in various forms of Kaposi's sarcoma. Lancet.

[B3] Antman K, Chang Y (2000). Kaposi's Sarcoma. New Engl J Med.

[B4] Koon HB, Bubley GJ, Pantanowitz L, Masiello D, Smith B, Crosby K, Proper J, Weeden W, Miller TE, Chatis P, Egorin MJ, Tahan SR, Dezube BJ (2004). Imatinib-Induced Regression of AIDS-Related Kaposi's Sarcoma. J Clin Oncol.

[B5] Stallone G, Schena A, Infante B, Di Paolo S, Loverre A, Maggio G, Ranieri E, Gesualdo L, Schena FP, Grandaliano G (2005). Sirolimus for Kaposi's sarcoma in renal-transplant recipients. N Engl J Med.

[B6] Quintas-Cardama A, Kantarjian H, O'Brien S, Garcia-Manero G, Rios MB, Talpaz M, Cortes J (2004). Granulocyte-colony-stimulating factor (filgrastim) may overcome imatinib-induced neutropenia in patients with chronic-phase chronic myelogenous leukemia. Cancer.

[B7] Volkow P, Zinser J (2000). Long-term remission of Kaposi's sarcoma in AIDS patients after ganciclovir therapy. Proceedings of ASCO Vol.

[B8] Chang Y, Parsonnet J Microbes, Malignancies (1999). KSHV, Kaposi's Sarcoma, and related lymphoproliferative disorders. Infection as a cause of human cancers.

[B9] Trattner A, Hodak E, David M, Sandbank M (1993). The appearance of Kaposi sarcoma during corticosteroid therapy. Cancer.

[B10] Guo WX, Antakly T, Cadotte M, Kachra Z, Kunkel L, Masood R, Gill P (1996). Expression and cytokine regulation of glucocorticoid receptors in Kaposi's sarcoma. Am J Pathol.

[B11] Guo WX, Antakly T (1995). AIDS-related Kaposi's sarcoma: evidence for direct stimulatory effect of glucocorticoid on cell proliferation. Am J Pathol.

[B12] Trattner A, Hodak E, David M, Neeman A, Sandbank M (1993). Kaposi's sarcoma with visceral involvement after intraarticular and epidural injections of corticosteroids. J Am Acad Dermatol.

[B13] Bruet A, Mahe A, Sei JF, Mathe C, Felsenheld C, Lechevalier L, Fendler JP (1990). Kaposi's sarcoma complicating long-term corticotherapy for severe asthma. Rev Med Interne.

[B14] Rybojad M, Borradori L, Verola O, Zeller J, Puissant A, Morel P (1990). Non-AIDS-associated Kaposi's sarcoma (classical and endemic African types):treatment with low doses of recombinant interferon-alpha. J Invest Dermatol.

[B15] Shimizu S, Tanaka M, Niizeki H, Miyakawa S, Ishiko A, Shimizu H (1995). Classic (non-AIDS-related) Kaposi's sarcoma in a Japanese patient, successfully treated with alpha-2b-interferon. Br J Dermatol.

[B16] Aldenhoven M, Barlo NP, Sanders CJ (2006). Therapeutic strategies for epidemic Kaposi's sarcoma. Int J STD AIDS.

[B17] Campistol JM, Gutiérrez-Dalmau A, Torregrosa JV (2004). Conversion to sirolimus: a successful treatment for posttransplantation Kaposi's sarcoma. Transplantation.

[B18] Zmonarski SC, Boratynska M, Rabczynski J, Kazimierczak K, Klinger M (2005). Regression of Kaposi's sarcoma in renal graft recipients after conversion to sirolimus treatment. Transplant Proc.

